# Hemodialysis with end-stage renal disease did not raise the risk of intracranial hemorrhage after a head injury

**DOI:** 10.1186/s13049-015-0168-1

**Published:** 2015-10-28

**Authors:** Hsin-Hung Chen, Chien-Chin Hsu, Shih-Feng Weng, Hung-Jung Lin, Jhi-Joung Wang, How-Ran Guo, Shih-Bin Su, Chien-Cheng Huang, Jiann-Hwa Chen

**Affiliations:** Department of Emergency Medicine, Chi-Mei Medical Center, 901 Zhonghua Road, Yongkang District, Tainan City, 710 Taiwan; Department of Biotechnology, Southern Taiwan University of Science and Technology, Tainan, Taiwan; Department of Healthcare Administration and Medical Informatics, Kaohsiung Medical University, Kaohsiung, Taiwan; Department of Emergency Medicine, Taipei Medical University, Taipei, Taiwan; Department of Medical Research, Chi-Mei Medical Center, Tainan, Taiwan; Department of Environmental and Occupational Health, College of Medicine, National Cheng Kung University, Tainan, Taiwan; Department of Occupational and Environmental Medicine, National Cheng Kung University Hospital, Tainan, Taiwan; Department of Occupational Medicine, Chi-Mei Medical Center, Tainan, Taiwan; Department of Leisure, Recreation and Tourism Management, Southern Taiwan University of Science and Technology, Tainan, Taiwan; Department of Medical Research, Chi Mei Medical Center, Liouying, Tainan, Taiwan; Department of Child Care and Education, Southern Taiwan University of Science and Technology, Tainan, Taiwan; Department of Geriatrics and Gerontology, Chi-Mei Medical Center, Tainan, Taiwan; Department of Emergency Medicine, Cathay General Hospital, No. 280, Sec. 4, Ren’ai Road, Da’an District, Taipei City, 106 Taiwan; Fu Jen Catholic University School of Medicine, Taipei, Taiwan

**Keywords:** End-stage renal disease, Head CT, Head injury, Hemodialysis, Intracranial hemorrhage

## Abstract

**Background:**

Hemodialysis (HD) treatment for end-stage renal disease (ESRD) (HD^+ESRD^) may increase the risk of intracranial hemorrhage (ICH) after a head injury (HI) for which heparin is used. However, the results of noncontrast head computed tomography (CT) in such patients are not always clear. We aimed to evaluate the effect of HD on the risk of ICH in ESRD patients and in controls without ESRD with HD (HD^−ESRD^), and to determine whether to lower the threshold of head CT in HD^+ESRD^ patients after HI.

**Methods:**

In this nationwide population-based study using Taiwan’s National Health Insurance Research Database, we enrolled 6938 HD^+ESRD^ HI patients for the case group and 13,876 randomly selected HD^−ESRD^ HI patients for the control group. Measures of the post-HI association between HD^+ESRD^ and ICH determined using conditional logistic regression.

**Results:**

Five hundred sixty-eight (2.74 %) patients had post-HI ICH: 185 in the HD^+ESRD^ group (2.67 % of cases) and 383 were from the HD^−ESRD^ group (2.76 % of controls). Conditional logistic regression analysis revealed that after adjusting for age, gender, diabetes, hypertension, congestive heart failure, stroke, cancer, and liver disease, HD^+ESRD^ patients had no higher odds of ICH (adjusted odds ratio [AOR]: 0.91; 95 % confidence interval [CI]: 0.75–1.11) than did HD^−ESRD^ patients. In the subgroup analysis of immediate ICH, HD^+ESRD^ patients had lower odds than did HD^−ESRD^ patients (AOR: 0.73; 95 % CI: 0.56–0.94).

**Conclusions:**

HD^+ESRD^ did not increase the post-HI risk of ICH. Therefore, it may not be necessary to lower the threshold of head CT in HD^+ESRD^ patients.

## Introduction

The population of patients with end-stage renal disease (ESRD) who require dialysis is progressively growing, and the mortality rate of this group is much higher than that of the general population in the U.S.[1]. In 2009, 116,395 patients started therapy for ESRD, and the prevalent population reached 571,414, including 398,861 dialysis patients [1]. The incident rate increased 1.1 %, to 355.4 per million, and total expenditures reached $42.5 billion [1]. In Taiwan, the number of ESRD patients who require hemodialysis (HD) has also increased considerably over the past two decades [2]. Taiwan has had the greatest incidence of ESRD since 2000, according to an international comparison based on data from the U.S. Renal Data System [3].

Head injury (HI) often results in lifelong physical, cognitive, behavioral, and social dysfunction for patients who, in turn, may place major social and financial burdens on their families and society [4, 5]. It is estimated that, in the U.S., around 5.3 million people are living with a HI-related disability [6], and in the E.U., approximately 7.7 million people who have experienced HI have disabilities [7]. There has been a shift toward older patients with HI for whom falls are the primary cause of HI among the elderly, resulting in more contusion injuries [8]. The high incidence of comorbidities and the frequent use of platelet aggregation inhibitors and oral anticoagulants among older patients have a negative influence on outcome after HI [8].

The widespread availability of head computed tomography (CT) has greatly helped physicians to better manage patients with HI [9]. Head CT is considered mandatory for all HI patients with an initial or persistent altered level of consciousness [9]. The role of the head CT in a patient with mild HI and a normal level of consciousness remains controversial [9]. In the New Orleans [10] and Canadian CT Clinical Decision Rules [11], head CT is indicated in patients with “known coagulopathy” who are on chronic anticoagulant therapy or are alcohol dependent; however, coagulopathy is potentially a significant risk factor for traumatic intracranial hemorrhage (ICH). HD and continuous renal replacement therapies require extracorporeal blood flow. Some type of anticoagulant, usually heparin or warfarin, is required to prevent thrombosis. However, there is no agreement about how heparin affects with HD^+ESRD^ patients with HI. We analyzed a population-based cohort taken from Taiwan’s National Health Insurance Research Database (NHIRD) to determine the risk of ICH after HI in HD^+ESRD^ patients. We hypothesized that HD^+ESRD^ increase the risk for ICH in patients with HI.

## Methods

### Data sources

Taiwan’s universal National Health Insurance (NHI) Program covers nearly 100 % of the country’s population [12]. The National Health Insurance Research Database (NHIRD), one of the largest and most complete population-based healthcare datasets in the world, contains encrypted patient identification numbers, ICD-9-CM codes for basic sociodemographic information, procedures, diagnoses, prescribed drugs, and dates of discharge and admission [13]. All the expenses of ESRD, HD, HI, and ICH are covered by NHI.

### Ethics statement

This study was conducted according to the Declaration of Helsinki. The Institutional Review Board at Chi-Mei Medical Center approved this study (IRB approved number 10404-E2) and waived the need for informed consents from the patients because the data in this study consists of unidentifiable, national, secondary data released to the public for research. The rights and welfare of the patients are not affected adversely by the waiver.

### Selection of cases and controls

**Fig. 1:**
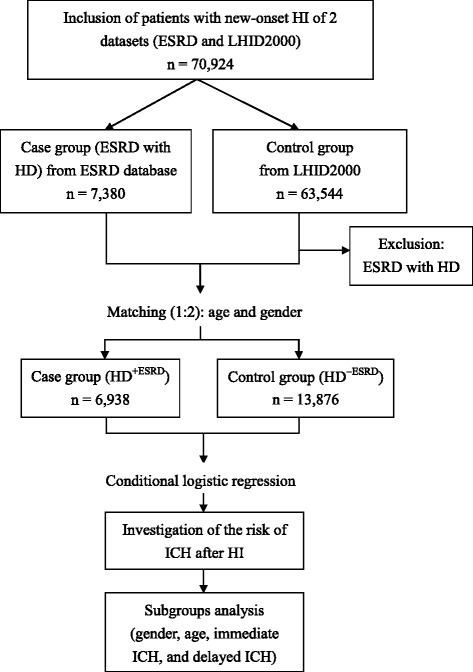
Flow chart of the study. HI, head injury; ESRD, end-stage renal disease; LHID2000, Longitudinal Health Insurance Database 2000; HD, hemodialysis; ICH, intracranial hemorrhage

This longitudinal study selected, for the case group, all ESRD patients on maintenance HD (HD^+ESRD^) with new-onset HI (ICD-9 codes: 850 [Concussion], 852 [Subarachnoid, subdural and extradural haemorrhage, following injury], 853 [Other and unspecified intracranial hemorrhage following injury], 854 [Intracranial injury of other and unspecified nature], 959.0 [Injury to head, face and neck], 959.01 [Head injury, unspecified], 959.09 [Injury of face and neck]) between 2002 and 2008 (Fig. 1). Their ESRD diagnoses were confirmed if the database indicated that they had a catastrophic illness certificate with the ICD-9 code number 585 for chronic kidney disease.

We also selected two matched controls per case from the Longitudinal Health Insurance Database 2000 (LHID2000), a data subset of the NHIRD that contains claims data for one million beneficiaries randomly selected from the full NHIRD in 2000. There are no significant differences in age, gender, and healthcare costs between the LHID2000 sample group and all NHI enrollees (Fig. 1). The definition of controls was patients who did not have ESRD with HD (HD^-ESRD^). Controls (HD^−ESRD^) were matched with cases by age, gender, and index year. Patients diagnosed with ESRD were excluded. As with the cases, we assigned the first use of medical care during the index year as the index date for controls.

We linked to the diagnostic codes through the ambulatory and inpatient care claims databases of the NHI. Baseline comorbidities affecting ICH that may have presented before the index date were diabetes mellitus (DM) (ICD-9 code 250), hypertension (HTN) (ICD-9 codes 401–405), congestive heart failure (CHF) (ICD-9 codes 428), stroke (ICD-9 codes 430–438), cancer (ICD-9 codes 140–208), and liver disease (ICD-9 codes 571.2, 571.4, 571.5, 571.6, 456.0–456.2, 572.2–572.8). We counted these comorbid conditions if they occurred either in the inpatient setting or in 3 or more ambulatory care claims coded 12 months before the index medical care date.

### Exposure assessment

This study compared the risk of ICH between cases and controls. In this study, we identified ICH that included medical codes for (i) immediate ICH after HI (ICD-9 codes: 852, 853, 854); and (ii) delayed ICH within 7 days of HI (ICD-9 codes: 852, 853, 854, 430 [Subarachnoid hemorrhage], 431 [Intracerebral hemorrhage], 432 [Other and unspecified intracranial hemorrhage], 432.1 [Subdural hemorrhage], 432.9 [Unspecified intracranial hemorrhage]). By definition, all patients had not been previously diagnosed with ICH.

### Subgroup analysis

We analyzed the effect of HD^+ESRD^ for ICH in HI patients in the subgroups based on gender, age, immediate ICH, and delayed ICH. Elderly was defined as ≥ 65 years old (Table 2).

### Statistical analysis

The significance of the differences in baseline characteristics and comorbid variables between the two groups was evaluated using Pearson *χ*^2^ tests for categorical variables and Student’s *t* test for continuous variables. We used conditional logistic regression (based on age, gender, and index year) to examine the association of ESRD with HD and ICH after HI after the potential confounders of DM, HTN, CHF, stroke, cancer, and liver disease, measured before the index date, had been adjusted for. SAS 9.3.1 for Windows (SAS Institute, Cary, NC, USA) was used for all analyses. Significance was set at *P* < 0.05.

## Results

### Demographic data of total patients with HI

Between 2002 and 2008, we recruited 6938 patients with HD^+ESRD^ and 13,876 age- and gender-matched controls, after ineligible patients had been excluded (Fig. 1; Table 1). The mean ages in the case and control groups on the HI date were identical: 65.52 ± 12.96 years old (Table 1). All patients were subclassified into 2 age groups: 0–64 years old and ≥ 65 years old (Table 1). Pearson *χ*^2^ tests revealed a significant difference between the distribution of the comorbidities of DM, HTN, CHF, stroke, cancer, and liver disease in cases and controls after matching (Table 1).

**Table 1:** Demographic characteristics of patients with head injury

Characteristic	Cases (HD^+ESRD^)	Controls (HD^−ESRD^)	*P*
Number of patients	6938	13,876	
Age (years)	65.52 ± 12.96	65.52 ± 12.96	0.9978
0–64	3004 (43.30)	6008 (43.30)	>0.999
≥ 65	3934 (56.70)	7868 (56.70)	
Gender			
Male	3040 (43.82)	6080 (43.82)	> 0.999
Female	3898 (56.18)	7796 (56.18)	
Comorbidities			
DM	3009 (43.37)	2538 (18.29)	< 0.0001
HTN	4056 (58.46)	5051 (36.40)	< 0.0001
CHF	1035 (14.92)	485 (3.50)	< 0.0001
Stroke	1276 (18.39)	1743 (12.56)	< 0.0001
Cancer	516 (7.44)	540 (3.89)	< 0.0001
Liver disease	727 (10.48)	939 (6.77)	< 0.0001

Data are presented as n (%) or means ± standard deviation. *ESRD* end-stage renal disease, *HD* hemodialysis, *DM* diabetes mellitus, *HTN* hypertension, *CHF* congestive heart failure

### Risk of ICH after HI

Of the 6938 HD^+ESRD^ patients, 185 (2.67 %) had ICH after HI during the follow-up period (Table 2). In the HD^−ESRD^ group, 383 of 13,876 patients (2.76 %) had ICH after HI during the follow-up period (Table 2). The crude OR was 0.97 (95 % CI: 0.81–1.15). After adjusting for patient age, gender, and comorbidities, HD^+ESRD^ was not associated with an increased risk of ICH after HI compared with the control group (AOR: 0.91; 95 % CI: 0.75–1.11) (Table 2).

**Table 2:** Comparison of the risk of intracranial hemorrhage after head injury between Case (HD^+ESRD^) and Control (HD^−ESRD^) groups

Outcome	Cases	Controls	Crude OR	P	AOR	P
Overall						
Yes, n (%)	185 (2.67)	383 (2.76)	0.97 (0.81–1.15)	0.6961	0.90 (0.74–1.10)	0.3012
No, n (%)	6753 (97.33)	13,493 (97.24)	1.00		1.00	
Gender						
Male						
Yes, n (%)	93 (3.06)	170 (2.80)	1.10 (0.85–1.42)	0.4788	1.03 (0.76–1.40)	0.8448
No, n (%)	2947 (96.94)	5910 (97.20)	1.00		1.00	
Female						
Yes, n (%)	92 (2.36)	213 (2.73)	0.86 (0.67–1.10)	0.2361	0.78 (0.59–1.03)	0.0826
No, n (%)	3806 (97.64)	7583 (97.27)	1.00		1.00	
Age (years)						
0 < 65						
Yes, n (%)	84 (2.80)	157 (2.61)	1.07 (0.82–1.40)	0.6112	1.01 (0.72–1.42)	0.9587
No, n (%)	2920 (97.20)	5851 (97.39)	1.00		1.00	
≥ 65						
Yes, n (%)	101 (2.57)	226 (2.87)	0.89 (0.70–1.13)	0.3430	0.83 (0.64–1.08)	0.1596
No, n (%)	3833 (97.43)	7642 (97.13)	1.00		1.00	

Data are presented as n (%). Conditional logistical regression was used. Adjusted by DM, HTN, CHF, stroke, cancer, and liver disease. *ESRD* end-stage renal disease, *HD* hemodialysis, *OR* odds ratio, *AOR* adjusted odds ratio

### Subgroup analysis

When patients were categorized by gender and age, the difference in the risk of ICH between 2 subgroups was not significant (Table 2). In an analysis of immediate and delayed ICH, HD^+ESRD^ patients had a lower risk than did HD^−ESRD^ patients (AOR: 0.73; 95 % CI: 0.56–0.94), especially in the females (AOR: 0.61; 95 % CI: 0.43–0.87) and those ≥ 65 years old (AOR: 0.70; 95 % CI: 0.50–0.97) (Table 3). In the delayed ICH analysis, there was no difference between HD^+ESRD^ and HD^−ESRD^ patients (Table 4).

**Table 3:** Comparison of the risk of immediate intracranial hemorrhage after head injury between Case (HD^+ESRD^) and Control (HD^−ESRD^) groups

Outcome	Case	Control	Crude OR	P	AOR	P
Overall						
Yes, n (%)	107 (1.54)	275 (1.98)	0.78 (0.62–0.97)	0.0263	0.73 (0.56–0.94)	0.0166
No, n (%)	6831 (98.46)	13,601 (98.02)	1.00		1.00	
Gender						
Male						
Yes, n (%)	56 (1.84)	118 (1.94)	0.95 (0.69–1.31)	0.7449	0.85 (0.57–1.28)	0.4420
No, n (%)	2984 (98.16)	5962 (98.06)	1.00		1.00	
Female						
Yes, n (%)	51 (1.31)	157 (2.01)	0.65 (0.47–0.89)	0.0070	0.61 (0.43–0.87)	0.0064
No, n (%)	3847 (98.69)	7639 (97.99)	1.00		1.00	
Age (years)						
0 < 65						
Yes, n (%)	46 (1.53)	116 (1.93)	0.79 (0.56–1.11)	0.1780	0.79 (0.51–1.22)	0.2860
No, n (%)	2958 (98.47)	5892 (98.07)	1.00		1.00	
≥ 65						
Yes, n (%)	61 (1.55)	159 (2.02)	0.77 (0.57–1.03)	0.0766	0.70 (0.50–0.97)	0.0314
No, n (%)	3873 (98.45)	7709 (97.98)	1.00		1.00	

Data are presented as n (%). Conditional logistical regression was used. Adjusted by DM, HTN, CHF, stroke, cancer, and liver disease. *ESRD* end-stage renal disease, *HD* hemodialysis, *OR* odds ratio, *AOR* adjusted odds ratio

**Table 4:** Comparison of the risk of delayed intracranial hemorrhage after head injury between the Case (HD^+ESRD^) and Control (HD^−ESRD^) groups

Outcome	Case	Control	Crude OR	P	AOR	P
Overall						
Yes, n (%)	78 (1.12)	108 (0.78)	1.46 (1.09–1.96)	0.0124	1.35 (0.97–1.88)	0.0737
No, n (%)	6860 (98.88)	13,768 (99.22)	1.00		1.00	
Gender						
Male						
Yes, n (%)	37 (1.22)	52 (0.86)	1.43 (0.94–2.19)	0.0990	1.41 (0.86–2.29)	0.1706
No, n (%)	3003 (98.78)	6028 (99.14)	1.00		1.00	
Female						
Yes, n (%)	41 (1.05)	56 (0.72)	1.48 (0.98–2.24)	0.0595	1.28 (0.81–2.02)	0.2950
No, n (%)	3857 (98.95)	7740 (99.28)	1.00		1.00	
Age (years)						
0 < 65						
Yes, n (%)	38 (1.26)	41 (0.68)	1.89 (1.21–2.96)	0.0055	1.46 (0.78–2.73)	0.2353
No, n (%)	2966 (98.74)	5967 (99.32)	1.00		1.00	
≥ 65						
Yes, n (%)	40 (1.02)	67 (0.85)	1.20 (0.81–1.78)	0.3703	1.16 (0.77–1.76)	0.4829
No, n (%)	3894 (99.98)	7801 (99.15)	1.00		1.00	

Data are presented as n (%). Conditional logistical regression was used. Adjusted by DM, HTN, CHF, stroke, cancer, and liver disease. *ESRD* end-stage renal disease, *HD* hemodialysis, *OR* odds ratio, *AOR* adjusted odds ratio

## Discussion

Using a nationwide population-based study design with a large sample, we found that ESRD with HD did not increase the risk of ICH after HI, regardless of age or gender. Moreover, HD^+ESRD^ patients had a lower risk of immediate ICH. To the best of our knowledge, this is the first study to report the associations between HD^+ESRD^ and the risk of ICH after HI. Our findings suggest that it may not be necessary for physicians to lower the threshold of head CT in HD^+ESRD^ patients after HI. In the HD^+ESRD^ elderly, a subgroup more sensitive to ICH, the criteria for head CT need not be different from those of the general population. This evidence should be valuable for establishing future guidelines for managing HI and may reduce medical costs by reducing the number of head CTs done, especially in the elderly.

Heparin has a short half-life of about one hour [14], which might be the most important reason that the risk for ICH does not rise after HI. One meta-analysis [15] suggests that using heparin use during dialysis does not significantly increase the risk of bleeding. Another study [16] reported that access-related bleeding was the most common in patients on HD [16]. Major bleeding related to heparin such as brain, gastrointestinal tract, and pericardium are uncommon, except for patient-specific reasons [16]. Heparin is commonly administered as a bolus injection before dialysis begins, and is then continuously infused during the dialysis treatment [17]. Before the end of dialysis, heparin infusions are typically stopped for approximately 30 min [16]. This procedure permits prompt hemostasis after the access needles have been removed [16]. It also protects patients from being exposed to a bleeding risk after HD.

There are no published studies about ICH after HI in HD^+ESRD^ patients. However, the published studies about the risk of non-traumatic ICH in HD patients have controversial results. In one Japanese study [18], HTN and the amount of HD prescribed were the risk factors, but anticoagulation with heparin was not. A study based on the US Renal Data System [19] showed that the occurrence rate of non-traumatic subdural hematoma in long-term HD patients is 10 times higher than that of the general population. The author concluded that it may be related to a greater use of anticoagulants in long-term HD patients; however, the study focused only on subdural hematoma and did not include patients with other types of ICH, such as subarachnoid, intracerebral, and epidural hemorrhages. In addition, it enrolled patients with traumatic and with non-traumatic ICH, which is different from our goal for traumatic ICH.

Bleeding is also a great concern in HD patients who need surgery. However, studies on this topic are also inconsistent. In two studies [20, 21], there was no greater incidence of bleeding in dialysis patients undergoing a vitrectomy for diabetic retinopathy. In a study about non-cardiac surgeries [22], HD patients had a risk for postoperative bleeding 1.4 times higher than that of non-HD patients. Although there are no randomized prospective studies, it is unlikely that individuals whose ESRD is well managed with HD would have a greater risk for clinically significant bleeding, even from multiple dental extractions.

Our study showed that the incidence of ICH in elderly HD^+ESRD^ patients was no higher than in elderly HD^−ESRD^ patients. Age is a risk factor for ICH, even in mild HI. In the Canadian CT Head Rule [11] and New Orleans Criteria [10], the elderly have a greater risk of clinically significant lesions that require acute neurosurgical intervention or prolonged inpatient observation [23, 24]. Early CT scanning, where it is available, is strongly indicated [23, 24].

Our study showed that HD^+ESRD^ patients had more comorbidities but a lower risk of immediate ICH after HI than did HD^-ESRD^ patients. Immediate ICH after HI always comes from a more severe trauma mechanism [25, 26]. HD^+ESRD^ patients are more sedentary than HD^-ESRD^ patients due to lowered hemoglobin level, lower extremity muscle strength, and poor physical functioning [27]. Therefore, more sedentary life style may contribute to a lower risk of immediate ICH after HI in HD^+ESRD^ patients.

There are several limitations to this study. First, there was no information on the severity of ICH or HI; therefore, we were unable evaluate the severity association between them. Second, some important drugs such as warfarin and aspirin were not investigated; therefore, we were unable to adjust for these variables as contributing factors in this study. Third, some HD^+ESRD^ patients used heparin-free protocol during HD. However, the proportion was small [28] and might not affect the final result of this large-scale study. Finally, despite our database being national, our findings may not be generalizable to other nations.

## Conclusions

This is the first nationwide population-based study to clarify that HD^+ESRD^ patients have no greater risk of ICH after HI than do HD^−ESRD^ patients, regardless of age. In the immediate ICH analysis, HD^+ESRD^ patients showed a lower risk than did HD^−ESRD^ patients. Therefore, it may not necessary for physicians to lower the threshold of head CT in HD^+ESRD^ patients after HI.
